# Impact of the COVID-19 pandemic on surgical outcomes in patients undergoing colorectal cancer surgery: A retrospective study and meta-analysis of data from 11,082 participants

**DOI:** 10.3389/fpubh.2022.907571

**Published:** 2022-09-29

**Authors:** Gang Tang, Feng Pi, Jie Tao, Zhengqiang Wei

**Affiliations:** ^1^Department of Gastrointestinal Surgery, The First Affiliated Hospital of Chongqing Medical University, Chongqing, China; ^2^Department of Hepatobiliary Surgery, The First Affiliated Hospital of Chongqing Medical University, Chongqing, China

**Keywords:** public health emergency, COVID-19, colorectal surgery, meta-analysis, surgical outcomes

## Abstract

**Background:**

The COVID-19 pandemic is affecting the care of patients with colorectal cancer worldwide, resulting in the postponement of many colorectal cancer surgeries. However, the effectiveness and safety of performing colorectal cancer surgery during the COVID-19 pandemic is unknown. This study evaluated the impact of the COVID−19 pandemic on surgical outcomes in patients undergoing colorectal cancer surgery.

**Methods:**

We retrospectively identified patients undergoing colorectal cancer surgery in January 21, 2019, to April 1, 2019, vs. January 21, 2020, to April 1, 2020. Data regarding perioperative outcomes (postoperative complications, conversion rate, duration of surgery, intraoperative blood loss, transfusion, reoperation, intensive care, histological examination, morbidity, and length of hospital stay) were retrieved and compared between the two cohorts. A meta-analysis of 14 studies was also conducted to assess the impact of the COVID−19 pandemic on surgical outcomes in patients undergoing colorectal cancer surgery.

**Results:**

The sample included 68 patients who underwent surgery in 2020 and 136 patients who underwent surgery in 2019. No patient was converted from laparoscopy to laparotomy or required reoperation. R0 resection was completed in all patients in both groups. There was no significant difference in postoperative complications (*p* = 0.508), duration of surgery (*p* = 0.519), intraoperative blood loss (*p* = 0.148), transfusion (0.217), intensive care (*p* = 0.379), mean lymph node yield (*p* = 0.205), vascular positivity rate (*p* = 0.273), nerve invasion rate (*p* = 0.713), anastomosis leak rate (*p* = 1), morbidity (*p* = 0.478), and length of hospital stay (*p* = 0.623) between the two groups. The meta-analysis also showed no significant difference in short-term outcomes between the two groups.

**Conclusions:**

Our study shows that the COVID-19 pandemic has not led to a deterioration in the surgical outcomes of colorectal cancer surgery or reduction in the quality of cancer removal. Therefore, we do not recommend postponing elective colorectal cancer surgery during the COVID-19 pandemic.

## Introduction

Coronavirus disease 2019 (COVID-19), the disease caused by severe acute respiratory syndrome coronavirus 2 (SARS-CoV-2) infection, has developed into a global pandemic since it was first reported in Wuhan in December 2019 (1). SARS-CoV-2 spread at a very fast speed, resulting in the global shortage of medical and health resources (2, 3). As of September 2, 2022, 601,189,435 confirmed cases and 6,475,346 deaths from COVID-19 have been reported (4). The median cost of treating a symptomatic case of COVID-19 in the United States is as high as US $3,045 (5). Governments have had to reallocate healthcare resources to ease the strain on healthcare systems caused by the COVID-19 pandemic, and this has led to many countries being forced to delay or cancel elective surgical treatment (1, 6). A multicenter survey from Italy showed that the COVID-19 epidemic has led to reduction in surgical activity in approximately 70% of medical units (7). Non-urgent and non-cancer operations were suspended, and some anesthesiologists, nurses, and surgeons were asked to help treat COVID-19 patients (7). Sozutek et al. reported that close to 50% of the medical resources, including intensive care unit (ICU) beds, in a large tertiary hospital in Turkey were used to treat patients with COVID-19 (8).

Colorectal cancer is the third most common cancer worldwide. It is estimated that 1,880,000 new cases occur yearly, with more than 900,000 deaths (9). Surgery is the main treatment for colorectal cancer (10). It is estimated that in the United States alone, a 4-months delay in colon cancer surgery could result in an additional 10,043 stage I to III deaths over a period of 5 years (11). Several studies have shown that patients with colorectal cancer are associated with increased risk and severity of COVID-19, suggesting that patients with colorectal cancer delay surgical treatment within 3 months of diagnosis (12). The 5-year survival rate for stage I and II colorectal cancer can reach 85%, whereas this rate is <40% for stage III and IV colorectal cancer (13). Delays in treatment often result in colorectal cancer being treated at a more advanced stage, which leads to more patients developing colorectal cancer-related complications, including intestinal obstruction and perforation, that require emergency surgical intervention (14, 15). Previous literature has shown that emergency surgical treatment is associated with increased morbidity and mortality after colorectal cancer surgery (14). In addition, delayed treatment of colorectal cancer not only leads to shorter survival, but also increases medical costs (16–18). A retrospective study of 6,936 colorectal cancer patients showed that longer waiting times significantly increased health care costs compared with shorter waiting times (19). However, it is unclear whether continuing standard colorectal cancer surgery during the COVID-19 pandemic increases morbidity and mortality.

Therefore, we conducted a retrospective cohort study to assess the impact of the COVID-19 epidemic on in-hospital mortality, postoperative complications, and surgical resection effectiveness in patients undergoing colorectal cancer surgery. In addition, we performed a meta-analysis of all previous studies that assessed the impact of the COVID-19 pandemic on colorectal cancer surgery and combined the results of this trial.

## Materials and methods

### Study design

This study was approved by Ethics Committee of The First Affiliated Hospital of Chongqing Medical University (2022–k427). We retrospectively enrolled consecutive patients who underwent surgery for colorectal cancer at The First Affiliated Hospital of Chongqing Medical University during the pandemic (group B; from January 21, 2020, to April 1, 2020), and a control group of colorectal cancer patients who underwent surgery during the same period in 2019 (group A; from January 21, 2019, to April 1, 2019).

Data on patients' characteristics, localization of the tumor, the use of neoadjuvant therapy, surgical procedures, conversion rate, disease stage, histological examination, anastomosis leak rate, morbidity, postoperative complications, and length of hospital stay were retrieved from patient charts for each patient.

### Outcomes

The primary endpoint was postoperative complications. Secondary endpoints included conversion rate, duration of surgery, intraoperative blood loss, transfusion, reoperation, intensive care, histological examination, anastomosis leak rate, morbidity, and length of hospital stay.

### Statistical analysis

Continuous variables are reported as mean (standard deviation) and categorical variables as number (percentage). Differences between operative data in the two time periods were tested with the independent-samples *t*-test for continuous variables and the chi-squared test for categorical data. All *p*-values were two-sided, and a *p*-value of <0.05 was considered statistically significant. All statistical analyses were conducted using IBM SPSS, version 27.0 (IBM, Armonk, New York, United States).

### Meta-analysis

A meta-analysis of cohort studies and case-control studies assessed the impact of the COVID−19 pandemic on short-term outcomes in patients undergoing colorectal cancer surgery and evaluated the external validity of our results. We followed the Preferred Reporting Items for Systemic Reviews and Meta-Analyses statement (CRD42022302596). The Embase, Web of Science, and PubMed databases were searched from inception to January 12, 2022. To be included, the studies had to meet the following criteria: (a) patients underwent colorectal cancer surgery; (b) patients underwent surgery during the COVID-19 pandemic; (c) patients underwent colorectal cancer surgery during the same period prior to 2020; (d) outcomes included any of the following: postoperative complications, postoperative mortality, ICU demand rate, laparoscopic convert to open, mean lymph node yield, R1 resections, and the hospital stay length; and (e) cohort study or case-control study. The following data were extracted from each study: first author, year, country, study design, sample, age, gender, primary disease, comparison date, and outcomes. The quality of included studies was assessed independently by two authors (Tang and Pi) based on the Newcastle-Ottawa Scale. The I^2^ statistic was used to evaluate the heterogeneity between studies. The mean differences or odds ratios (ORs) for individual studies were combined using a random effects meta-analysis when I^2^ was >50 %. Otherwise, the fixed-effect model was selected (20). Sensitivity analysis was performed using the exclusion of one study to assess the effect of each study on the total effect size. Analyses were conducted using Review Manager (RevMan) Version 5.3 (The Nordic Cochrane Center, The Cochrane Collaboration 2014; Copenhagen, Denmark). A *p*-value of <0.05 was considered statistically significant.

## Results

### Baseline characteristics

A total of 204 patients (125 male and 79 female) who underwent surgery for colorectal cancer were included. There was 50% reduction in the number of patients who underwent surgery during the pandemic (136 procedures in 2019 vs. 68 procedures in 2020). The mean age of the 204 colorectal cancer patients was 61.69 ± 12.31 years, and the average body mass index (BMI) was 22.94 ± 2.76 kg/m2. A total of 178 (87.25%) patients were covered by local resident medical insurance and 26 (12.75%) were covered by non-local medical insurance. No significant difference was found in terms of sex, age, BMI, ASA grade, tumor stage, comorbidity, and type of medical insurance between the 68 patients who underwent surgery during the COVID-19 pandemic and the 136 patients who underwent surgery in 2019.

There were 10 cases of emergency surgery (7.4%) and 126 cases of elective surgery (92.6%) in group A, and six (8.8%) cases of emergency surgery and 62 (91.2%) cases of elective surgery in group B. There was no significant difference between the two groups in the proportion of patients undergoing emergency surgery (*p* = 0.713). The incidence of preoperative ileus was similar (*p* = 0.836) in both groups.

Laparoscopic and laparotomy procedures was conducted in 121 (89%) and 15 patients (11%), respectively in group A, while eight patients (11.8%) underwent laparotomy and 60 patients (88.2%) underwent laparoscopic surgery in group B. There were no differences in the use of laparoscopic approach (*p* = 0.876). The rates of left colon, right colon, and rectal cancers were similar between groups A and B (*p* = 0.420). There was no difference (*p* = 0.295) in the proportion of patients receiving neoadjuvant therapy between groups (10 and eight patients in groups A and B, respectively). The basic characteristics of patients are summarized in Table 1.

**Table 1:** Basic characteristics of patients.

	**Group A (2019 *n* = 136)**	**Group B (2020 *n* = 68)**	***P* value**
Age (years), $\bar{x}$ ± s	62.00 ± 12.51	61.07 ± 11.94	0.613
**Gender (%)**			0.684
Male	82 (60.3)	43 (63.2)	
Female	54 (39.7)	25 (36.8)	
BMI, $\bar{x}$ ± s	23.07 ± 2.98	22.67 ± 2.24	0.294
Local residents (%)	118 (86.8)	60 (88.2)	0.767
Emergency cases (%)	10 (7.4)	6 (8.8)	0.713
Preoperative Ileus (%)	48 (35.3)	25 (36.8)	0.836
Diabetes mellitus (%)	12 (8.8)	4 (5.9)	0.461
Hypertension (%)	35 (25.7)	12 (33.8)	0.196
ASA Grade, $\bar{x}$ ± s	2.52 ± 0.56	2.50 ± 0.56	0.790
Neoadjuvant therapy received (%)	10 (7.4)	8 (11.8)	0.295
**Treatment modality (%)**			0.876
Open	15 (11.0)	8 (11.8)	
Laparoscopy	121 (89.0)	60 (88.2)	
**Tumor location (%)**			0.420
Right colon	36 (26.5)	13 (19.1)	
Left colon	35 (25.7)	22 (32.4)	
Rectum	65 (47.8)	33 (48.5)	
**UICC stage (%)**			0.455
I	23 (16.9)	16 (23.5)	
II	62 (45.6)	27 (39.7)	
III	37 (27.2)	21 (30.93)	
IV	14 (10.3)	4 (5.9)	

### Surgical results

Among all patients, 181 (88.7%) patients underwent laparoscopic surgery, and the remaining 23 (11.3%) underwent laparotomy. No patient was converted from laparoscopy to laparotomy. In addition, 21 (30.9%) and 34 (25.0%) patients underwent protective ostomy in 2020 and 2019, respectively. Table 2 shows a similar rate of protective stoma in the two groups (*p* = 0.372).

**Table 2:** Clinical parameters of patients who receive curative resection at the same period of 2019 and 2020.

	**Group A (2019 *n* = 136)**	**Group B (2020 *n* = 68)**	***P* value**
Duration of surgery (min), $\bar{x}$ ± s	218.47 ± 87.99	210.43 ± 75.08	0.519
intraoperative blood loss, $\bar{x}$ ± s	92 ± 100.57	72.1 ± 74.22	0.148
Conversions (%)	0 (0)	0 (0)	
Transfusion (%)	3 (2.2)	0 (0)	0.217
Preventive stoma (%)	34 (25.0)	21 (30.9)	0.372
Postoperative complications (%)	36 (26.5)	21 (30.9)	0.508
Reoperation (%)	0 (0)	0 (0)	
Mortality (%)	1 (0.7)	0 (0)	0.478
Intensive care (%)	5 (3.8)	1 (1.5)	0.379
Preoperative waiting (days), $\bar{x}$ ± s	6.46 ± 3.57	5.18 ± 3.60	0.016
Postoperative stay (days), $\bar{x}$ ±s	8.41 ± 2.99	9.37 ± 3.77	0.050
Hospital stay (days), $\bar{x}$ ± s	14.87 ± 4.51	14.55 ± 4.54	0.623
Mean Lymph node yield, $\bar{x}$ ± s	16.46 ± 9.46	14.85 ± 6.22	0.205
R1 Resections (%)	0 (0)	0 (0)	
Perineural invasion (%)	10 (7.4)	6 (8.8)	0.713
Lymphovascular invasion (%)	9 (6.6)	2 (2.9)	0.273

There was no significant difference in surgical duration between groups A and B (*p* = 0.519). The amount of intraoperative blood loss (*p* = 0.148) and incidence of intraoperative blood transfusion (0.217) were similar in the two groups. The incidence of total postoperative complications was similar: 26.5% in group A vs. 30.9% in group B (*p* = 0.508). In addition, the incidence of anastomotic leakage was similar (*p* = 1.0) in groups A (1.5%) and B (1.5%). None of the patients in either group required reoperation. Only one death was reported in group A, and there was no significant difference (*p* = 0.478) in mortality between the two groups. There were five patients and one patient in group A and B, respectively, who required ICU treatment, and this difference was not statistically significant (*p* = 0.379).

During the COVID-19 pandemic, the average hospital stay and the average hospital stay after surgery were 14.55 ± 4.54 days and 9.37 ± 3.77 days, respectively. No significant differences (*p* = 0.623; *p* = 0.050) were observed between groups A and B. However, the average length of hospital stay before surgery during the COVID-19 pandemic was shorter (*p* = 0.016) than before the COVID-19 pandemic.

The mean number of lymph nodes harvested in group A and B was 16.46 ± 9.46 and 14.85 ± 6.22, respectively, with no significant difference between the two groups (*p* = 0.205). R0 resection was completed in all patients in both groups. During the COVID-19 pandemic, the incidence of vascular positivity and nerve invasion was 2.9 and 8.8%, respectively, and these rates were not significantly different to pre-COVID-19 rates (*p* = 0.273; *p* = 0.713).

### Meta-analysis

The literature search yielded 1,349 potentially eligible records, of which 46 articles were reviewed in full. In addition to our study, 13 studies (1, 12, 21–31) published between 2020 and 2022 were included for analysis. The details of the 14 eligible studies are presented in Table 3. The risk of bias was assessed as low in all 14 included studies (Table 3).

**Table 3:** Characteristics of trials included in the meta-analysis.

**References**	**Country**	**Study design**	**Sample**	**Age**	**Gender (M/ F)**	**Primary disease**	**Comparison date**	**Outcomes**	**NOS**
Ferahman et al. (22)	Turkey	Retrospective case-control study	I: 35 C: 27	I: 61 C: 65	I: 22/33 C: 17/10	Colorectal cancer	Between March and June 2020 vs. between March and June 2019	Hospital stay, Anastomosis leak, Mortality, morbidity	7
Cui et al. (21)	China	Retrospective case-control study	I: 67 C: 101	I: 67 C: 67	I: 44/23 C: 44/57	Colorectal cancer	February 1 to May 31, 2020 vs. February 1 to May 31 in 2019	Postoperative morbidity, Conversion, Hospital stay, Lymph node harvested, Mortality	8
Merchant et al. (23)	United Kingdom	Prospective cohort study	I: 47 C: 33	I: NA C: NA	I: NA C: NA	Colorectal cancer	During the 11 weeks following the national UK lockdown on 23rd March 2020 vs. the same time period in 2019	R1 Resections rate, Conversion rate, Mean Lymph node yield	9
Allaix et al. (12)	Italy	Retrospective case-control study	I: 44 C: 32	I: NA C: NA	I: NA C: NA	Colorectal cancer	March 9 and April 15, 2020 vs. the same time period in 2019	R1 Resections rate, Mean Lymph node yield	8
Rashid et al. (24)	United Kingdom	Retrospective case-control study	I: 22 C: 10	I: 74 C: 69	I: 16/6 C: 7/3	Colorectal cancer	Between 1 March 2020 and 30 April 2020 vs. the same time period in 2019	R1 Resections rate, Postoperative morbidity, Hospital stay, Mortality	7
Smith et al. (25)	Denmark	Retrospective cohort study	I: 681 C: 1176	I: 73 C: 72	I: NA C: NA	Colorectal cancer	From 1 March 2020 to 1 August 2020 vs. the same time period in 2019	Postoperative morbidity, Mortality	8
Tschann et al. (26)	Austria	Retrospective case-control study	I: 63 C: 71	I: 69 C: 66	I: 34/29 C: 42/29	Colorectal cancer	From 1 January 2020 to 31 December 2020 vs. the same time period in 2019	Postoperative morbidity, Mean Lymph node yield, Hospital stay	8
Uyan et al. (27)	Turkey	Retrospective case-control study	I: 48 C: 56	I: 63 C: 65	I: 31/17 C: 32/24	Colorectal cancer	Between March 11, 2020, and December 31, 2020 vs. the same time period in 2019	Postoperative morbidity, Mortality, Hospital stay	8
Williams et al. (28)	Australia	Multicentre retrospective cohort study	I: 1036 C: 2081	I: NA C: NA	I: 549/487 C: 1167/914	Colorectal cancer	During the second quartile (Q2—April to June) and forth quartile (Q4—October to December) 2020 vs. the same time period in 2019	Mortality	8
Xu et al. (29)	China	Retrospective case-control study	I: 710 C: 828	I: NA C: NA	I: 438/272 C: 518/310	Colorectal cancer	January 1, 2020 and May 3, 2020 vs. the same time period in 2019	Postoperative morbidity, Hospital stay	8
Yeung et al. (30)	United Kingdom	Prospective case-control study	I: 107 C: 117	I: NA C: NA	I: NA C: NA	Colorectal cancer	From 1st March to 31st June, 2020 vs. the same time period in 2019	Hospital stay	9
Losurdo et al. (31)	Italy	Retrospective case-control study	I: 118 C: 132	I: 78 C: 77	I: 57/61 C: 68/64	Colorectal cancer	March 2019 to March 2020 vs. April 2020 to April 2021	Postoperative morbidity	8
Rottoli et al. (1)	Italy	Retrospective case-control study y	I: 1481 C: 1755	I: 70 C: 70	I: 635/846 C: 753/1002	Colorectal cancer	March–December 2020 vs. the same time period in 2019	Postoperative morbidity, Mortality	8
Current study, 2022	China	Retrospective cohort study	I: 68 C: 136	I: 61 C: 62	I: 43/25 C: 82/54	Colorectal cancer	January 21, 2020 to April 1, 2020 vs. the same time period in 2019	Postoperative morbidity, Conversion, Hospital stay, Lymph node harvested, Mortality, Anastomotic leak, R1 Resections rate	8

C, control group; F, Female; I, intervention group; M, Male; NA, not available; UK, United Kingdom.

**Figure 1:**
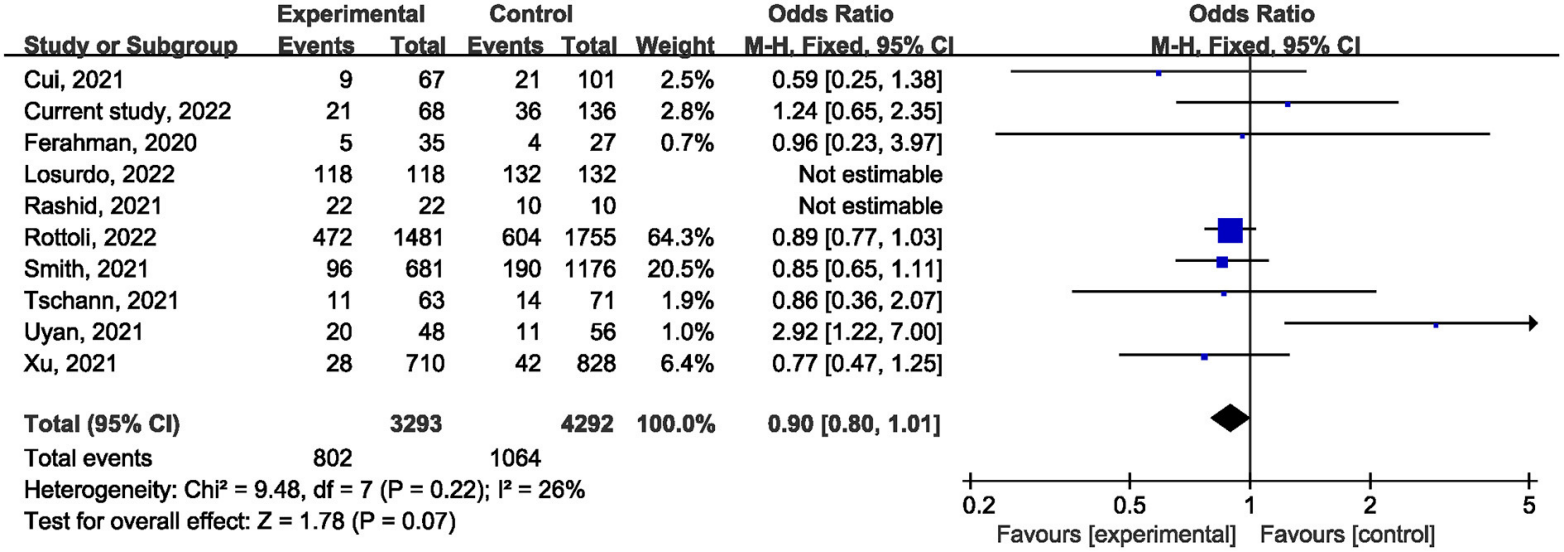
The impact of the COVID-19 pandemic on postoperative morbidity.

**Figure 2:**
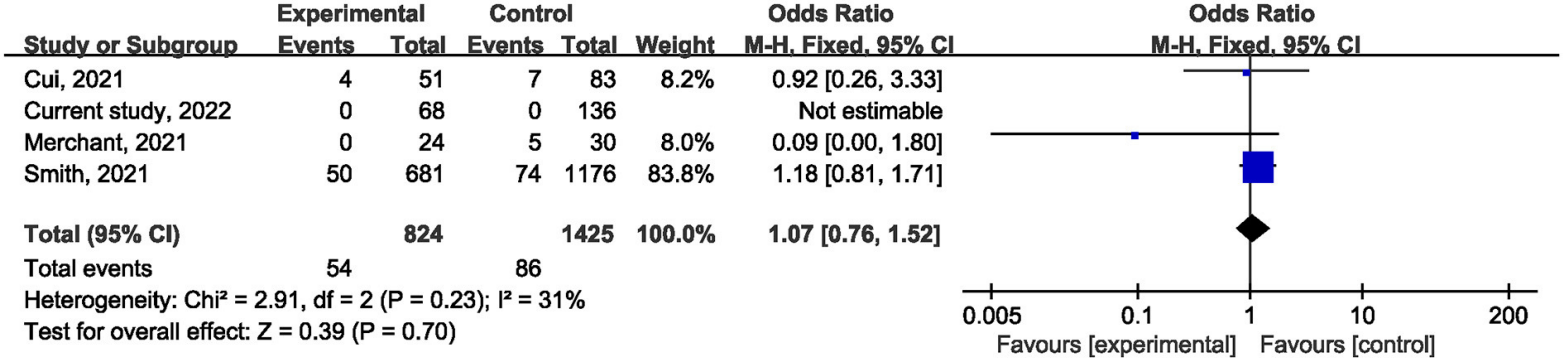
The impact of the COVID-19 pandemic on the conversion rate.

**Figure 3:**
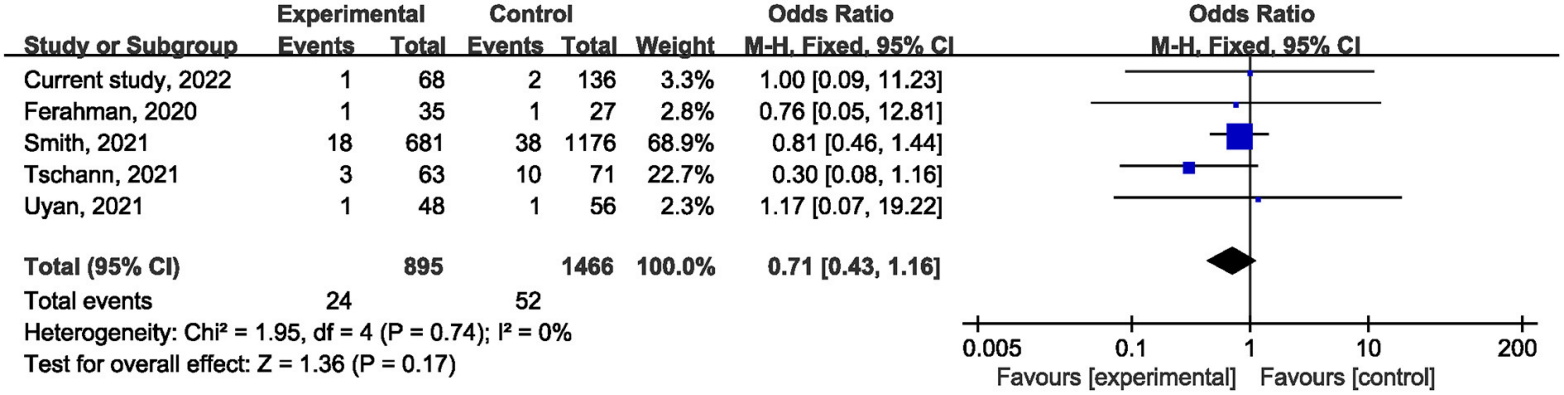
The impact of the COVID-19 pandemic on the incidence of anastomotic leakage.

**Figure 4:**
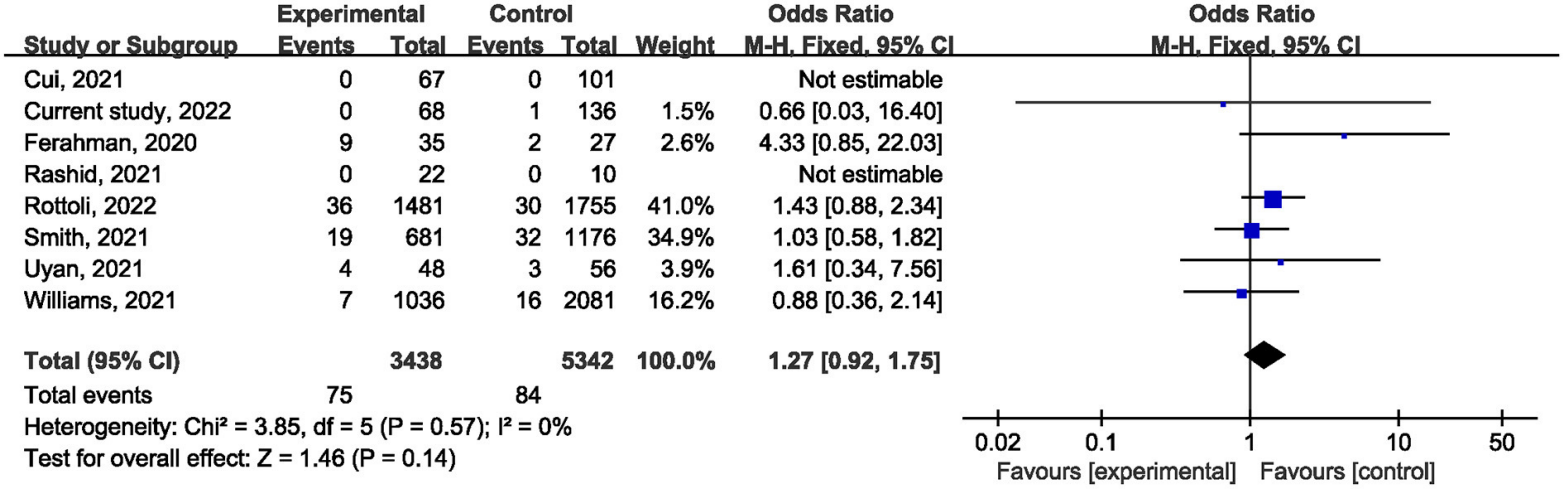
The impact of the COVID-19 pandemic on postoperative mortality.

**Figure 5:**
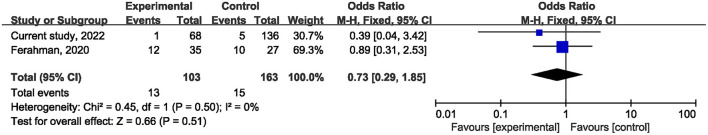
The impact of the COVID-19 pandemic on the intensive care unit demand rates.

**Figure 6:**
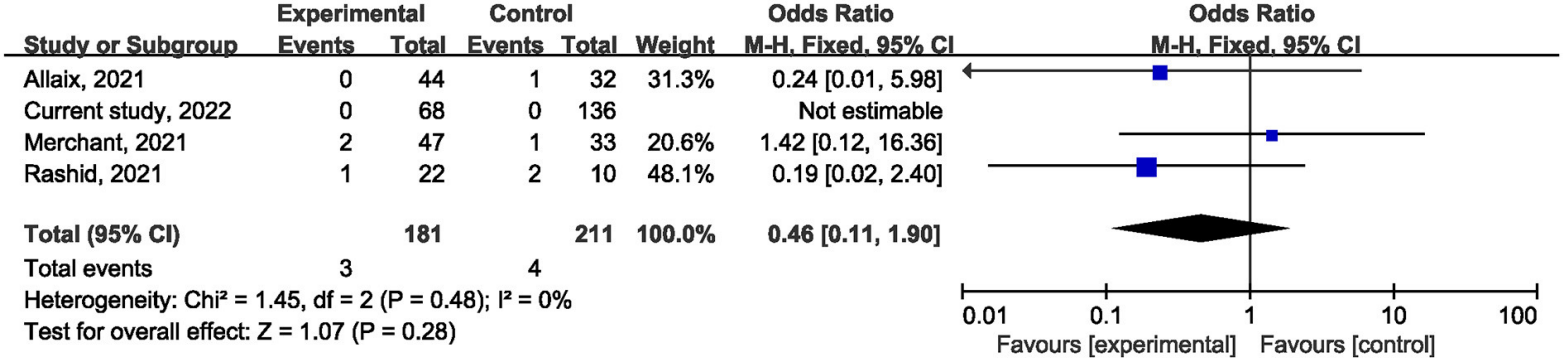
The impact of the COVID-19 pandemic on the R1 resections rate.

**Figure 7:**
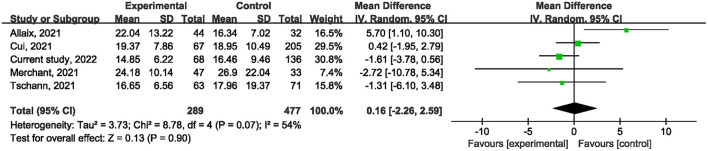
The impact of the COVID-19 pandemic on mean lymph node yield.

**Figure 8:**
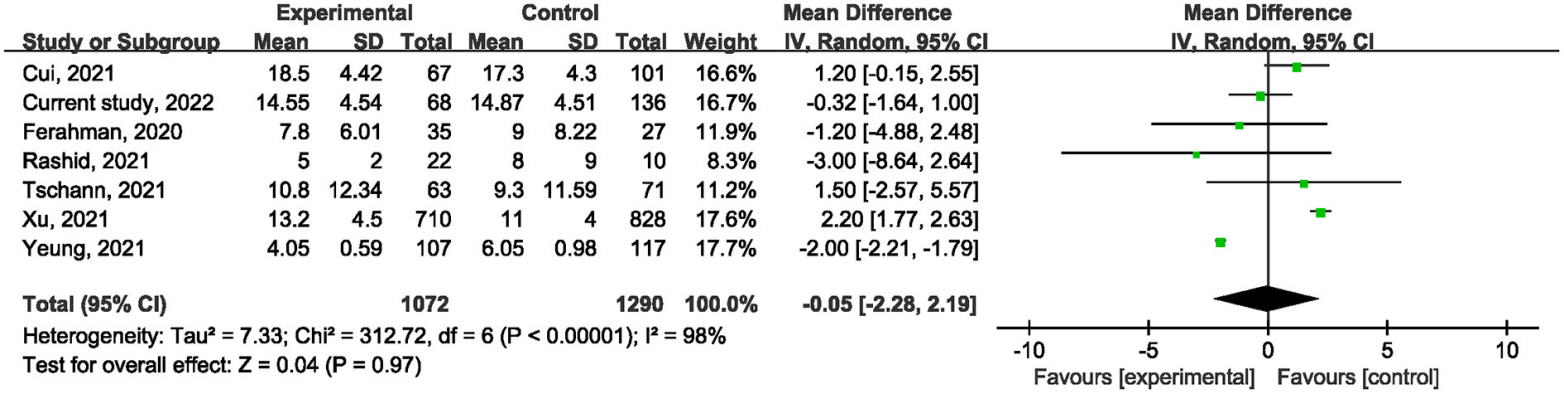
The impact of the COVID-19 pandemic on the length of hospital stay.

Ten studies (1, 21, 22, 24–27, 29, 31) reported on postoperative mortality. There was no statistically significant difference [OR, 0.90; 95% confidence interval (CI), 0.80, 1.01; *p* = 0.07] (Figure 1) in the overall incidence of postoperative complications between patients in the COVID-19 pandemic group and those in the pre-COVID-19 pandemic group, with low heterogeneity between studies (I^2^ = 26%, *p* = 0.22). Meta-analysis of the four studies (21, 23, 25) showed no significant difference with regard to conversion rate. The result was OR = 1.07; 95% CI, 0.76, 1.52; *p* = 0.70 (Figure 2) with high heterogeneity (I^2^ = 31%). Data on the anastomotic leakage rate were described in five studies (22, 25–27). When colorectal cancer surgery was performed during the COVID-19 pandemic, this did not increase the incidence of anastomotic leakage (OR, 0.71; 95% CI, 0.43, 1.16; *p* = 0.17; I^2^ = 0%) (Figure 3). The pooled effect sizes of the eight studies (1, 21, 22, 24, 25, 27, 28) showed no significant difference in mortality (OR, 1.27; 95% CI, 0.92, 1.75; *p* = 0.14; I^2^ = 0%) (Figure 4) between the two groups. Two studies (22) reported on ICU demand rate. There was no significant difference in the ICU demand rate (OR, 0.73; 95% CI, 0.29, 1.85; *p* = 0.51; I^2^ = 0%) (Figure 5) between the two groups. Four studies (12, 23, 24) described R1 resection rate. There were no significant differences in the R1 resection rate (OR, 0.46; 95% CI, 0.11, 1.90; *p* = 0.28; I^2^ = 0%) (Figure 6) for colorectal cancer surgery performed during the COVID-19 pandemic compared with that pre-pandemic. A meta-analysis of five studies (12, 21, 23, 26) did not show any significant differences in mean lymph node yield (MD, 0.16; 95% CI, −2.26, 2.59; *p* = 0.90; I^2^ = 54%) (Figure 7). Colorectal cancer surgery during the COVID-19 pandemic did not increase the length of hospital stay (MD, −0.05; 95% CI, −2.28, 2.19; *p* < 0.00001; I^2^ = 98%) (Figure 8) compared with that before the pandemic. The results of the sensitivity analysis showed that no single study significantly affected the overall effect size for postoperative mortality, conversion rate, mortality, ICU demand rate, R1 resection rate, anastomotic leakage rate, mean lymph node yield, and length of hospital stay. The total effect size for postoperative mortality changed (OR, 0.88; 95% CI, 0.78, 0.99; *p* = 0.03; I^2^ = 0%) when the study by Uyan et al. (27) was excluded.

## Discussion

Colorectal cancer is one of the most common cancers worldwide and the third leading cause of death among cancer patients. Early surgical intervention is the main strategy to improve the prognosis of colorectal cancer (22). The COVID-19 pandemic has posed a serious challenge to health systems around the world (12). Data from some studies suggest that surgery is associated with an increased risk of COVID-19 (12). In addition, patients with cancer are more likely to be infected with SARS-CoV-2 than those without cancer, and infection with SARS-CoV-2 leads to a worse prognosis (12, 32). Due to the COVID-19 pandemic, a large number of elective surgeries for colorectal cancer have had to be postponed (2, 12). A multicenter survey found that up to 61% of surgeons were prepared to defer elective colorectal cancer surgery, with 29% willing to delay for more than 2 months (6). Some elderly colorectal cancer patients are reluctant to go to hospitals for radical surgery due to lockdown policies and fear of contracting COVID-19 (22). Our study also found significant reduction in the number of patients undergoing radical colorectal cancer surgery during the COVID-19 pandemic compared with that in the same period in 2019. The decline in elective colorectal cancer surgery has been widely reported at home and abroad (21, 23, 25). Research by Williams et al. (28) found that there were fewer colorectal cancer operations during the COVID-19 pandemic than the historical average.

However, a recent meta-analysis suggested that delayed surgical treatment of more than 4 weeks is associated with poorer oncology outcomes in colorectal cancer (33). This has raised concerns among clinicians about the consequences of delaying elective colorectal cancer surgery, and some researchers have opposed delaying elective colorectal cancer surgery during the COVID-19 pandemic (12, 23). An observational study in South Korea showed that surgery for gastrointestinal cancer during a pandemic is safe with appropriate isolation measures and should not be postponed regardless of whether the cancer is early or advanced (8). Therefore, there is widespread concern about the safety and effectiveness of colorectal cancer surgery during the COVID-19 pandemic. Our surgical information showed that the COVID-19 pandemic had not resulted in an increase in surgical time or intraoperative blood loss. Comparison between the pandemic and pre-pandemic groups revealed no statistically significant differences in the epidemiological variables of this study (ASA grade, sex, age, BMI, comorbidity, neoadjuvant therapy, surgical method, tumor site and stage), indicating that the samples were similar and could be compared. In this study, length of hospital stay, postoperative mortality and morbidity were selected to evaluate the safety of elective colorectal cancer surgery during the pandemic, and R1 resection rate and average number of lymph nodes obtained were selected to evaluate the effectiveness of surgery. In terms of safety, we found that colorectal cancer surgery during the COVID-19 pandemic did not increase postoperative morbidity, mortality, or length of hospital stay compared with pre-COVID-19 pandemic surgery. In terms of effectiveness, R1 resection rate and mean number of lymph nodes obtained did not differ between the COVID-19 pandemic group and the pre-pandemic group. More importantly, our meta-analysis also confirmed that the COVID-19 pandemic does not affect the safety and effectiveness of colorectal cancer surgery. This has important clinical implications as we provide evidence that the COVID-19 pandemic has no impact on the safety and effectiveness of colorectal cancer surgery, which may reduce treatment delays in colorectal cancer surgery and thus improve outcomes for colorectal cancer patients. Similarly, He et al. (18) included patients who underwent radical colorectal cancer surgery between December 20, 2019 and March 20, 2020. They divided patients into pre-pandemic and post-pandemic groups (before and after January 20, 2020), and the results suggested that colorectal cancer surgery during the pandemic was safe. A retrospective cohort analysis by Vicente et al. (17) showed that colorectal cancer surgery during the COVID-19 pandemic did not increase postoperative morbidity and mortality, and they recommended that colorectal cancer surgery continue during the pandemic. Concerns that colorectal cancer surgery during a pandemic might increase the incidence of anastomotic leakage have led some researchers to propose an ostomy rather than a direct anastomosis. Both our study and our meta-analysis showed similar rates of anastomotic leakage during colorectal cancer surgery during the pandemic and during the pre-pandemic period. This suggests that the strategy of encouraging stomostomy is not advisable since stomostomy and closure may be associated with greater morbidity (25).

Chemotherapy is one of the most important adjuvant therapies for colorectal cancer. Studies have reported reduction in postoperative chemotherapy for colorectal cancer in India (34), Scotland (35), and the United Kingdom (23) during the COVID-19 pandemic. In Turkey (36), the incidence of delay in administration of chemotherapy increased from 7.6 to 50%. In China (37), the number of outpatient chemotherapy visits decreased by 17.1% during the COVID-19 epidemic compared with before the COVID-19 epidemic. Delayed chemotherapy results in poorer oncological outcomes (38). Therefore, choosing to postpone colorectal cancer surgery due to delayed or reduced chemotherapy may further impair the long-term prognosis of patients with colorectal cancer.

At present, the end of the COVID-19 pandemic is far in sight, and many regions are seeing recurrent outbreaks. While delaying elective surgery can help cope with the stress of COVID-19, it can jeopardize the survival of colorectal cancer patients and could expose surgeons to legal issues after the pandemic ends in the event of adverse tumor-related outcomes. We confirm that colorectal cancer surgery is safe and effective during the COVID-19 pandemic through our current study and meta-analysis involving 11,082 people, and we do not recommend delaying elective colorectal cancer surgery during the pandemic.

Our study had the following limitations. First, this study was a single-center retrospective study, which may be affected by some confounding factors, but the basic characteristics of the two groups included in this study were comparable. Second, the sample size of this study was limited, which may not be enough to prove a statistical difference between the groups. Therefore, in order to enhance statistical strength, we conducted a meta-analysis involving 11,082 people from 14 studies, which further confirmed the reliability of our results. Third, because the time-window is still narrow, our study might not include the full complement of patients who decided to delay elective surgery and presented later for emergency surgery. As such, the overall impact of COVID-19 remains to be quantified. Finally, due to limitations in follow-up time, we were unable to assess the long-term outcomes of colorectal cancer patients undergoing surgery during the COVID-19 pandemic, which needs to be further explored by future studies.

## Conclusion

The COVID-19 pandemic is affecting the management of patients with colorectal cancer worldwide, resulting in the postponement of colorectal cancer surgeries. The delay of surgery not only increases the medical costs, but also jeopardizes the long-term survival of patients. However, it is unclear whether performing colorectal cancer surgery during the COVID-19 pandemic increases morbidity and mortality. Therefore, a retrospective cohort study was conducted to assess the impact of the COVID-19 pandemic on in-hospital mortality, postoperative complications, and the effect of surgical resection in patients undergoing colorectal cancer surgery. In addition, we performed a meta-analysis of all previous studies that evaluated the impact of the COVID-19 pandemic on colorectal cancer surgery. Our study shows that colorectal cancer surgery performed during the COVID-19 pandemic does not increase postoperative morbidity and mortality, and more importantly, the R1 resection rate and average number of lymph nodes identified during the pandemic were comparable to those performed before the pandemic. Therefore, we do not recommend postponing elective colorectal cancer surgery during the COVID-19 pandemic.

## Data availability statement

The original contributions presented in the study are included in the article/supplementary material, further inquiries can be directed to the corresponding author.

## Ethics statement

The studies involving human participants were reviewed and approved by Ethics Committee of the First Affiliated Hospital of Chongqing Medical University. The patients/participants provided their written informed consent to participate in this study.

## Author contributions

Conceptualization and primary responsibility for final content: ZW, GT, JT, and FP. Data collection, analyses, and writing—original draft preparation: JT, GT, and FP. Writing—review and editing: ZW, GT, and FP. All authors contributed to the article and approved the submitted version.

## Funding

This study was funded by Chongqing joint medical scientific research project of science and health (2018ZDXM007) and Chongqing key diseases Research and Application Demonstration Program (No. 2019ZX003).

## Conflict of interest

The authors declare that the research was conducted in the absence of any commercial or financial relationships that could be construed as a potential conflict of interest.

## Publisher's note

All claims expressed in this article are solely those of the authors and do not necessarily represent those of their affiliated organizations, or those of the publisher, the editors and the reviewers. Any product that may be evaluated in this article, or claim that may be made by its manufacturer, is not guaranteed or endorsed by the publisher.
